# The Burning Question

**Published:** 1913-07

**Authors:** 


### The Burning Question.

A SEX WAR.—The latest development in the suffrage movement in England substantiates the opinion advanced by some publicists that we are on the eve of an actual state of war between the sexes. The militant leaders have so worked themselves into a psychological frenzy that reason can no longer be appealed to. They have so excited the younger women by their immoderate harangues that the movement has assumed the aspect of a fanatical outbreak. They have wantonly broken the constitutional law and insulted the dignity of woman, until all respect and consideration due her sex has been forfeited. They have placed themselves in the criminal class and the spectacle of men and officials having to defend their lives and homes against an army of women by force of arms is a disgrace to our twentieth century civilization. It is very evident that such volcanic agitation, such vituperation, such despicable conduct and womanly humiliation would not be displayed in the cause of equal franchise. Something more potential, more fundamental, must lie at the bottom of such a universal and vehement protest.

That cause is the burning question of modern femininism—whether men and women are physically, socially and ethically equal!

Enlightened womanhood feels that society has dealt harshly with her; that she bears a greater burden than the opposite sex, though she is physically the weaker; that her ethical nature has been insulted and her heart's promptings starved and mistrusted. She has learned with horror that the marriage bed, so blissfully trusted, is too often the prelude to invalidism, lost hope and a wasted life. She has seen the inhumanity of man, and indignantly resents the marriage rite as a cloak for the satisfaction of loveless passion. She shrinks from the early deterioration of her physical strength and beauty because of excessive child-bearing, and she vehemently protests against the wrong society imposes by her ostracism for the same acts which are condoned in man. She indignantly resents the sexual bondage that thousands of her sisters are subjected to because of our social and economic conditions. These and many more reasons arising from the sex relation, she professes to think, will be corrected by the right of the ballot.

But suffrage is a mere subterfuge for the real issue. Whether it obtains or not makes no difference. The emancipated woman will find little inclination to exercise her rights.

Why, then, this false pretense? Why not strike at the root of the evil? There is every reason for this protest; why mystify the truth?

To make a custom permanently and generally accepted necessitates the recasting of old sentiments—change of heart, so to speak—which requires time to accomplish. Laws regulating the personal actions of the individual are instinctively resisted, and it is only by an evolutionary process new concepts are formed.

The New Sentiment.—There is little wonder that this agitation should develop many phases to the question. The more knowing minds, who intuitively feel the deeper meaning involved, make little pretense to disclaim the growing tendency which looks upon the morality of the sex relation in a very different light. With these thinking people (for there are men as well as women that hold to this sentiment) the sex instinct is natural, and therefore it is pure. Hence the exercise of this function is pure—i. e., moral. They say, then, "Why any restraint to a natural impulse?" And they are led to believe that this attitude will free woman from the wrongs she now endures. They argue, further, that if exercise of the sexual function is necessary to the man, why not equally so to the woman, as she is endowed with the same instincts and desires as he? If she has squared her conscience with this belief, she feels herself in every sense a free woman, the equal with man, and is in the position to treat with him on her own terms.

Granting this situation to be true (and, indeed, so far as nature is concerned it is true), for, from the biological standpoint, "nature knows no immorality," there should be no distinction between ante-nuptial and extra-marital relations. But it is also true (which fact these advocates overlook) that man is endowed with other instincts besides the all-important one. Being natural, they are moral also. Then it follows that the exercise of any of their functions would not be a desecration of nature. For instance, the *moral* instinct is one of these, and it is given to man for the purpose of *conserving* the sexual instinct. It is the *inhibitory* impulse to this function. Every organ of the body has its stimulating and inhibitory nerves to properly regulate its functions. So with the sex instinct. It is the dissociation of these instincts that is responsible for our immorality. Therefore, when these radicals (for radicals they are) advocate "free love" (for such is the meaning of their doctrine), they place themselves in the attitude of holding to the truth, but not the whole truth. Again, they are in error in presuming that man and woman have the same necessity for sexual gratification. Physiologically, man is aggressive, and woman is receptive, and their needs are not the same. Otherwise there would be no attraction, one for the other. Where there is true affinity, one is as necessary as the other in forming an alliance, and in this particular lies the sexual equality of woman. We may conclude, therefore, that neither "militant tactics" nor "free love" is the solution for this situation.

THE HIGHER MORALITY.—The solution lies (if it were possible to be universal) in a love sublimated by the intellectual faculty. A normal, virile woman wants the love of a man for *herself, what she is,* and not for the passion her personality might have stimulated for the time being. If she feels this assurance, she will give the best that her heart and sympathy can bestow, and there would be no wrongs to square. To her, this means living up to the promptings of the heart, the grasping of what one's nature passionately desires. To her morality is greater liberty, and restraint is oppressive. She makes the distinction that morality is the freedom of love, and that unchastity is sexual relations without love. To her the marriage bond does not necessarily mean morality. On the contrary, the grossest immorality may be committed under the yoke of marriage. That is to say, the union of such souls is independent of marriage bonds, and is the highest morality. This ideal is exemplified in the life of Nora in Ibsen's "Doll House" and by Rahel as portrayed by Ellen Key. Rahel said: "The highest personal morality consists in being true—coercive marriage is the great social lie." Indeed, speaking in fundamental terms, *society* may be defined as sex-life regulated, and *culture* as sex-life sublimated.

But this individualism requires the highest development of the intellectual and psychological nature, to act as unfailing checks, in the exercise of such freedom. Can it become universal? While woman's love is fairly constant, in every walk of life, there are but few who have this *sublime love.* To the majority of women, love is but strong desire towards the opposite sex, to whom she looks for its fulfillment. To them, marriage is sexual morality protected by society, and to them there can never come the same freedom and joy that comes to their superior sisters. The world adores this superwoman. Man is like an aspen leaf before her matchless smile. *She is the salvation of the race;* without her ennobling influence nations would perish.

But if all humankind cannot be superwomen and supermen, what can society do to increase the sum of social happiness and efficiency? Obviously the elect need no restraint. But who would gainsay that the State should not impose proper restraints as a matter of expediency? As the permanency of the State is dependent on the moral quality, it should then be the first consideration of society to develop this attribute. This can be done by eradicating the superstition and prudery about sex and its relation to society; by teaching a clean, practical personal sex hygiene; by compelling responsibility for venereal diseases; by lessening the burdens of maternity; by allowing greater freedom of divorce for infelicity, and by relieving the burden of philanthropy through practical eugenics.

After all, the whole matter is one of progress and evolution. Are we progressing?

"She walketh veiled and sleeping,
For she knoweth not her power;
She obeyeth but the pleading
Of her heart, and the high leading
Of her soul, unto this hour.
Slow advancing, halting, creeping,
Comes the woman to this hour.
She walketh veiled and sleeping,
For she knoweth not her power."

